# The complete chloroplast genome of *Calophyllum membranaceum* Gander & Champ 1849 (Calophyllaceae, Malpighiales) and its phylogenetic analysis

**DOI:** 10.1080/23802359.2026.2622795

**Published:** 2026-02-08

**Authors:** Nhung Ngo Thi Cam, Chinee Surita Padasas-Adalla, Natsaranyatron Singharajkomron, Yihui Tan, Liangqiang Huang, Xinmin Pan, Jingling Li, Guoan Shen

**Affiliations:** ^a^ Institute of Medicinal Plant Development, Chinese Academy of Medical Sciences & Peking Union Medical College, Beijing, China; ^b^ NTT Hi-Tech Institute, Nguyen Tat Thanh University, Ho Chi Minh, Vietnam; ^c^ Nguyen Tat Thanh University Center for Hi-Tech Development, Ho Chi Minh City, VietNam; ^d^ Department of Biological Sciences, College of Arts and Sciences, Cavite State University, Indang, Philippines; ^e^ Department of Pharmacology and Physiology, Faculty of Pharmaceutical Sciences, Chulalongkorn University, Bangkok, Thailand; ^f^ Guangxi Hongyao Biotechnology Co., Ltd, Guangxi Zhuang Autonomous Region, Liuzhou City, China

**Keywords:** *Calophyllum membranaceum*, genome assembly, plastome, phylogenetic analysis

## Abstract

This study presents the first complete chloroplast genome assembly of *Calophyllum membranaceum* Gander & Champ 1849, a species noted for its pharmacologically active metabolites. The chloroplast genome of *C. membranaceum* measures 160,820 bp in length and an overall GC content of about 36.4%. It contains a total of 131 genes, which consisted of 86 protein-coding genes, 37 tRNAs, and 8 rRNAs. The phylogenetic analysis based on chloroplast data from the Malpighiales order suggests a close relationship between *C. membranaceum* and *C. brasiliense.* Our results provide valuable genomic resource that support future research on genetic resources, conservation, and medicine.

## Introduction

*Calophyllum membranaceum* Gander & Champ 1849, a species belonging to the Calophyllaceae family, is primarily found in tropical and subtropical regions of Southeast Asia. *C. membranaceum* gained attention due to its production of diverse metabolites. Numerous studies have shown that the genus *Calophyllum* produces various classes of secondary metabolites, including coumarins, glycosides, phenolics, flavonoids, steroids, and terpenoids, which are valuable for drug discovery and development (Gupta and Gupta 2020). Specifically, *C. membranaceum* is used in traditional medicine to treat conditions such as rheumatism, arthritis, lower back pain, and to promote wound healing (Zhu et al. 2018). Recent research has focused on isolating new compounds from the plant’s extracts, including chromanone acids (Shen et al. 2024), xanthones (Chen et al. 2011; Li et al. 2022; Yu et al. 2022) and C-glycosides (Zhu et al. 2018), some of which exhibit significant anti-inflammatory effects and transcriptional inhibitory activity against RXRα (Ming et al. 2016).

Despite its pharmaceutical potential, the chloroplast genome of *C. membranaceum* has not yet been described, representing a significant gap in understanding the species’ evolutionary relationships. Adding genetic data on this species is crucial for its classification, identification, and the conservation of biodiversity. In this study, we sequenced, assembled, and annotated the chloroplast genome of *C. membranaceum*. Additionally, we constructed a phylogenetic tree to provide a comprehensive view of the biological relationships within the Calophyllaceae family.

## Materials and methods

### Plant material, DNA extraction, and sequencing

**Figure 1.**
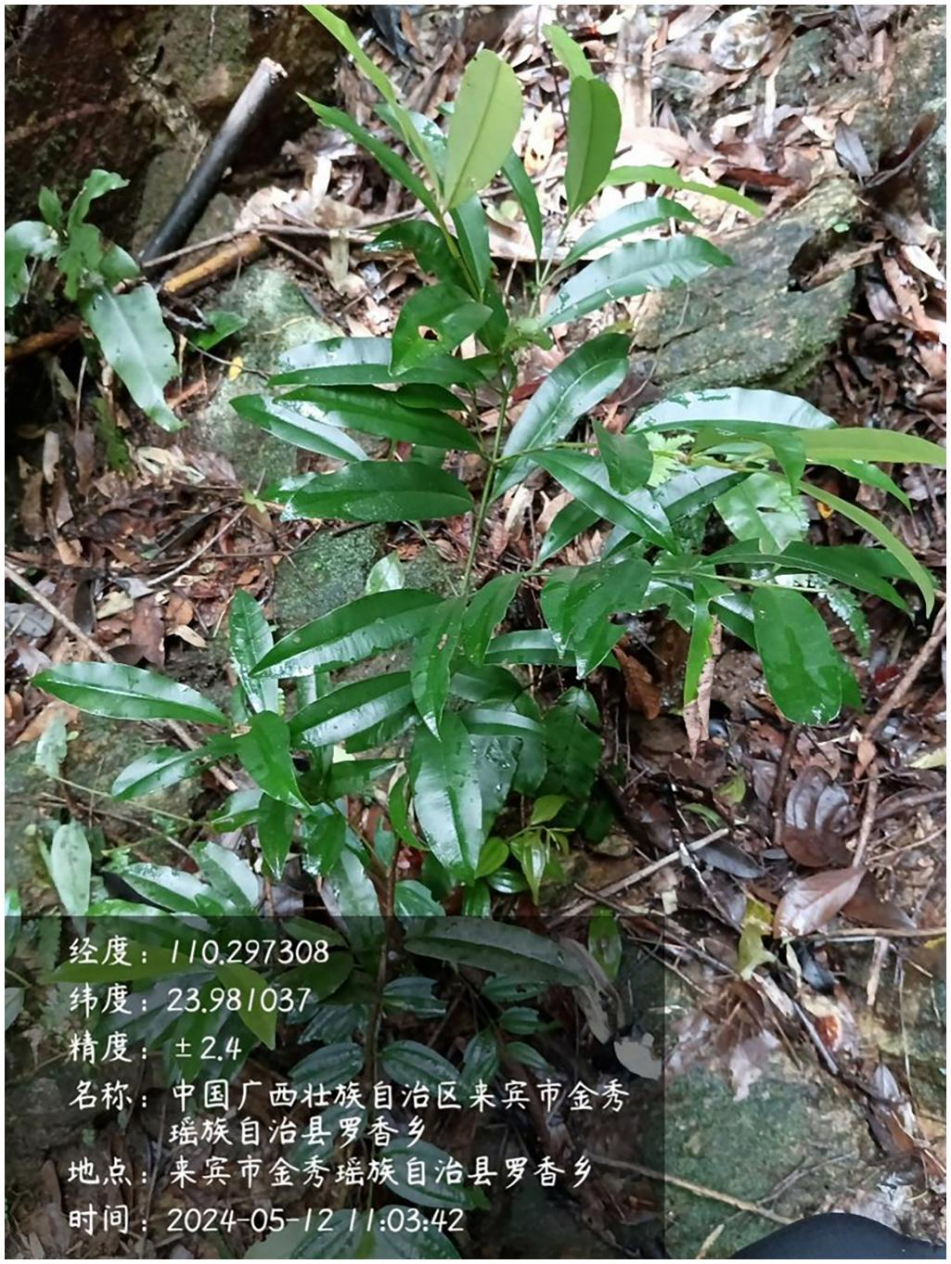
Photographs of *Calophyllum membranaceum*. This plant is a small tree or shrub-like; young branchlets are four-angled with narrow wings; leaves are thinly leathery, oblong or oblong-lanceolate, measuring 6–12 cm in length, with an acuminate, acute, or caudate apex and a cuneate base; petiole length 6–10 mm. The photo was taken by Xinmin Pan (one of authors). When the photo was taken, the camera app automatically recorded the location data (in Chinese) and marked it in the lower left corner. The information translate into English is as follows: **Longitude**: 110.297308; **Latitude**: 23.981037; **Precision**: ±2.4 m; **Site**: Luoxiang Township, Jinxiu Yao Autonomous County, Laibin City, Guangxi Zhuang Autonomous Region, China; **Location**: Luoxiang Township, Jinxiu Yao Autonomous County, Laibin City; **Time**: 2024-05-12 11:03:42.

The leaves of *C. membranaceum* used in this study were collected from Jinxiu county, Guangxi, China (23°98′N, 110°29′E) (Figure 1). A voucher specimen was identified by Dr. Haimei Chen (Institute of Medicinal Plant Development, Chinese Academy of Medical Sciences & Peking Union Medical College, hmchen@implad.ac.cn), and was registered at the Institute of Medicinal Plant Development, with the voucher number Y79 (contact: Guoan Shen, gashen@implad.ac.cn). Genomic DNA was isolated using the DNeasy Plant Mini Kit (Cat. 69104, Qiagen) and sequenced on the Illumina platform with a paired-end read length of 150 bp (Porebski et al. 1997). This process yielded high-quality reads that amounted to around 13.7 Gb of data. The complete chloroplast genome of *C. membranaceum* was assembled *de novo* using the GetOrganelle v1.7.7.1 tool (Jin et al. 2020b). Genome annotation was performed with CpGAVAS2 (Shi et al. 2019), and the annotated chloroplast genome was submitted to GenBank with accession numbers PQ463636. The circular chloroplast genome map, including *cis*-splicing and *trans*-splicing gene maps, was generated using the CPGview website (Liu et al. 2023).

### Phylogenetic analysis

To determine the phylogenetic position of *C. membranaceum,* eight chloroplast genome sequences from the Calophyllaceae family and 16 from the order Malpighiales were retrieved from the GenBank database. The list of these species is provided in Table S1. Protein-coding sequences from all sampled species were extracted and aligned using MAFFT v7 with default settings (Katoh and Standley 2013). Phylogenetic reconstruction was carried out using the maximum likelihood method, based on the TVM+I + G evolutionary model as determined by ModelFinder in PhyloSuite v1.2.3 (Kalyaanamoorthy et al. 2017). The phylogenetic tree was generated using the maximum likelihood approach, with 1,000 bootstrap replications, as implemented in PhyloSuite software (Zhang et al. 2020). *Klainedoxa gabonensis* was utilized as an outgroup. The final tree was modified and displayed utilizing the iTOL v5 web server (Letunic and Bork 2021).

## Results

**Figure 2.**
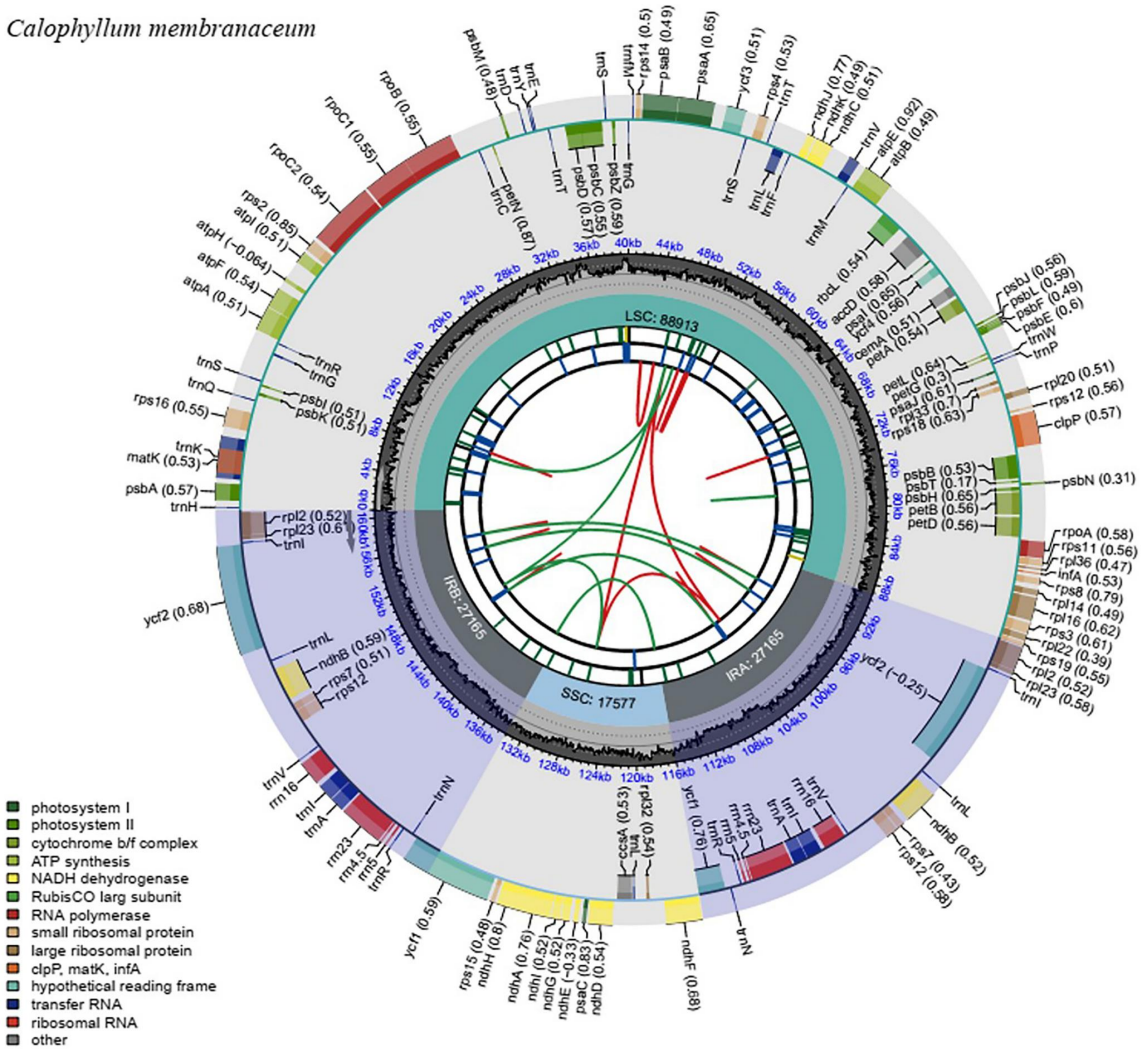
The circular diagram of the *Calophyllum membranaceum* chloroplast genome was produced utilizing CPGview. The genes shown outside of the circle are transcribed clockwise, while those insides are transcribed counterclockwise. Genes belonging to different functional groups are color-coded. The dashed area in the second inner circle indicates the GC content of the chloroplast genome. The four regions of the plastome—LSC, SSC, IRa, and IRb—are illustrated in the third circle, showing their respective sizes. The next circle highlights short tandem repeats, or microsatellite sequences, shown as short bars in various colors. Each color represents a specific type of repeat: black signifies complex repeats, while green, yellow, purple, blue, orange, and red indicate repeat unit sizes from 1 to 6. The five track represents long tandem repeats using short blue bars. The innermost circle displays dispersed repeats, linked by red arcs for forward repeats and green arcs for palindromic repeats.

The chloroplast (cp) genome of *C. membranaceum* was assembled *de novo* using 45,662,575 reads, resulting in an average coverage depth of 5735.73× and a minimum coverage of 489× (Figure S1). The plastome of *C. membranaceum* was characterized by a circular structure of 160,820 bp, comprising a large single-copy region (LSC) of 88,913 bp, a small single-copy region (SSC) of 17,577 bp, and two inverted repeats (IR), each measuring 27,165 bp (Figure 2). The overall GC content of the genome is approximately 36.4%, with the highest levels found in the IR regions at 42.3%. The GC content in the LSC and SSC regions is 34.0% and 30.6%, respectively.

In total, the cp genome contains 131 annotated genes, including 86 protein-coding genes (PCGs), 37 transfer RNA (tRNA) genes, and 8 ribosomal RNA (rRNA) genes. In the IR regions, 6 PCGs (*ndhB, rpl2, rpl23, rps7, rps12, ycf2, ycf1*), 4 rRNAs (*rrn5, rrna4.5, rrn23, rrn16*) and 7 tRNAs (*trnA-UGC, trnI-CAU, trnI-CAU, trnL-CAA, trnN-GUU, trnR-ACG, trnV-GAC*) are duplicated. Twelve genes (*rps16, atpF, rpoC1, ycf3, petB, petD, rpl16, rpl2* (×2)*, ndhB* (×2), and *ndhA*) each contain one intron, while two genes (*clpP* and *rps12*) possess two introns (Figure S2). Notably, *rps12* is recognized as a *trans*-spliced gene (Figure S3).

**Figure 3.**
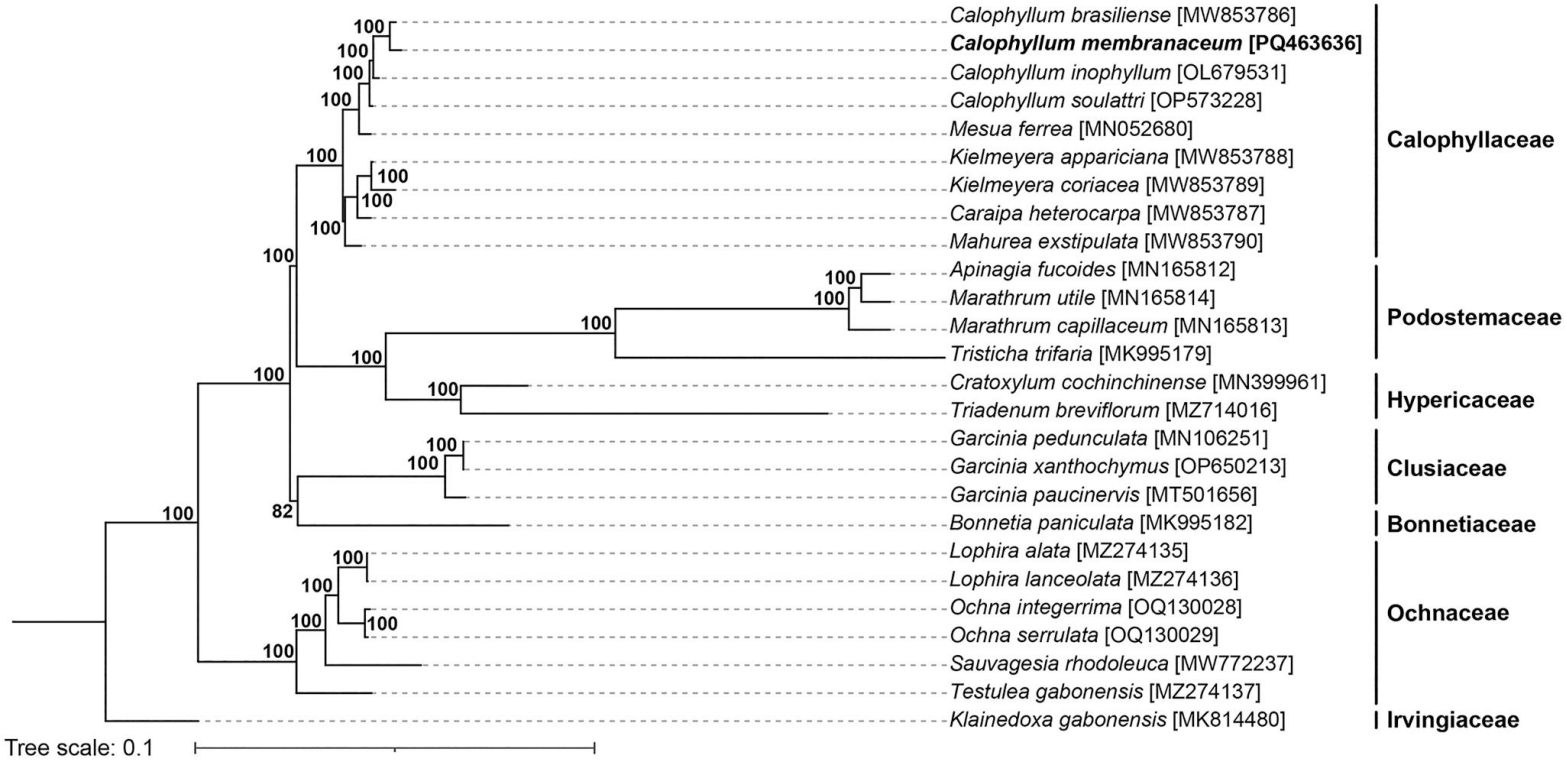
The phylogenetic tree was constructed using chloroplast protein-coding genes from 25 species within the Calophyllaceae family, employing the maximum likelihood method. Bootstrap support values are shown at each node, indicating the robustness of the inferred relationships. Bootstrap value of 100 was hidden. The sequences newly assembled in this study are highlighted in bold to distinguish them from previously published data: *Calophyllum brasiliense* (MW853786) (Trad et al. 2021), *Calophyllum inophyllum* (OL679531) (direct submission to NCBI), *Calophyllum soulattri* OP573228) (Pahayo et al. 2023b), *Mesua ferrea* (MN052680) (Wang et al. 2019), *Kielmeyera appariciana* (MW853788) (Trad et al. 2021), *Kielmeyera coriacea* (MW853789) (Trad et al. 2021), *Caraipa heterocarpa* (MW853787) (Trad et al. 2021), *Mahurea exstipulate* (MW853790) (Trad et al. 2021), *apinagia fucoides* (MN165812) (Bedoya et al. 2020), *Marathrum utile* (MN165814) (Bedoya et al. 2020), *Marathrum capillaceum* (MN165813)(Bedoya et al. 2020), *Tristicha trifaria* (MK995179) (Jin et al. 2020a), *Cratoxylum cochinchinense* (MN399961) (Jin et al. 2020a), *Triadenum breviflorum* (MZ714016) (Sudmoon et al. 2022), *garcinia pedunculata* (MN106251) (Yang et al. 2019), *Garcinia xanthochymus* (OP650213) (direct submission to NCBI), *Garcinia paucinervis* (MT501656) (direct submission to NCBI), *Bonnetia paniculata* (MK995182) (Jin et al. 2020a), *Lophira alata* (MZ274135) (Mascarello et al. 2021), *Lophira lanceolata* (MZ274136) (Mascarello et al. 2021), *Ochna integerrima* (OQ130028) (Nhat Nam et al. 2023), *Ochna serrulate* (OQ130029) (Nhat Nam et al. 2023), *Sauvagesia rhodoleuca* (MW772237) (direct submission to NCBI), *Testulea gabonensis* (MZ274137) (Mascarello et al. 2021), and *Klainedoxa gabonensis* (MK814480) (Jin et al. 2019). The tree was rooted using *Klainedoxa gabonensis* as the outgroup.

A phylogenetic tree was constructed using the maximum likelihood method, based on the coding sequences (CDS) from chloroplast genomes of seven families (Calophyllaceae, Podostemaceae, Hypericaceae, Clusiaceae, Bonnetiaceae, and Ochnaceae). All branches showed high bootstrap support (≥82%), indicating strong confidence in the relationships. The analysis revealed that the cp genome of *C. membranaceum* is closely related to that of *C. brasiliense* (Figure 3).

## Discussion and conclusion

This study presents the first chloroplast genome of *C. membranaceum,* providing its annotation and characteristic analysis. It establishes the phylogenetic position of *C. membranaceum* within the Calophyllaceae family, marking it as the ninth plastome characterized in this family. The cp genome of *C. membranaceum* is 160,820 bp in length, which is consistent with the size range found in this genus, from 160,253 bp in *C. brasiliense* to 161,381 bp in *C. soulattri* (Pahayo et al. 2023). The gene content is consistent with other *Calophyllum* species, containing 86 PCGs, 37 tRNAs, and 8 rRNAs (Pahayo et al. 2023). Among these genes, *ycf3* contains single intron, which is characteristic of the Calophyllaceae family, as noted in the study by Trad et al. (2021).

The phylogenetic tree indicates that species within the *Calophyllum* genus form a distinct clade, supported by a 100% bootstrap value, which is consistent with previous cp genome studies (Pahayo et al. 2023; Shi et al. 2024). Specifically, *C. membranaceum* is shown to be most closely related to its sister species, *C. brasiliense*. This finding is the first report on the phylogenetic relationship of *C. membranaceum* based on cp genome data.

The results of this study enhance the understanding of the cp genomic features of *C. membranaceum* and shed light on its evolutionary relationships within the Calophyllaceae family. This work also serves as a valuable reference for future studies on chloroplast genomes of related species in this family.

## Supplementary Material

Supplemental Material

Supplemental Material

Supplemental Material

## Data Availability

The genome sequence data that support the findings of this research are available in GenBank under the accession number PQ463636. The BioProject, BioSample, and SRA numbers are PRJNA1170470, SAMN44107503, and SRR30920838, respectively.
