# Supplementary figures and images for: The complete chloroplast genome of Calophyllum membranaceum Gander & Champ 1849 (Calophyllaceae, Malpighiales) and its phylogenetic analysis
Source: Mitochondrial DNA B Resour. 2026 Feb 8;11(3):378–82. doi: 10.1080/23802359.2026.2622795 (PMC12888349; doi:10.1080/23802359.2026.2622795)

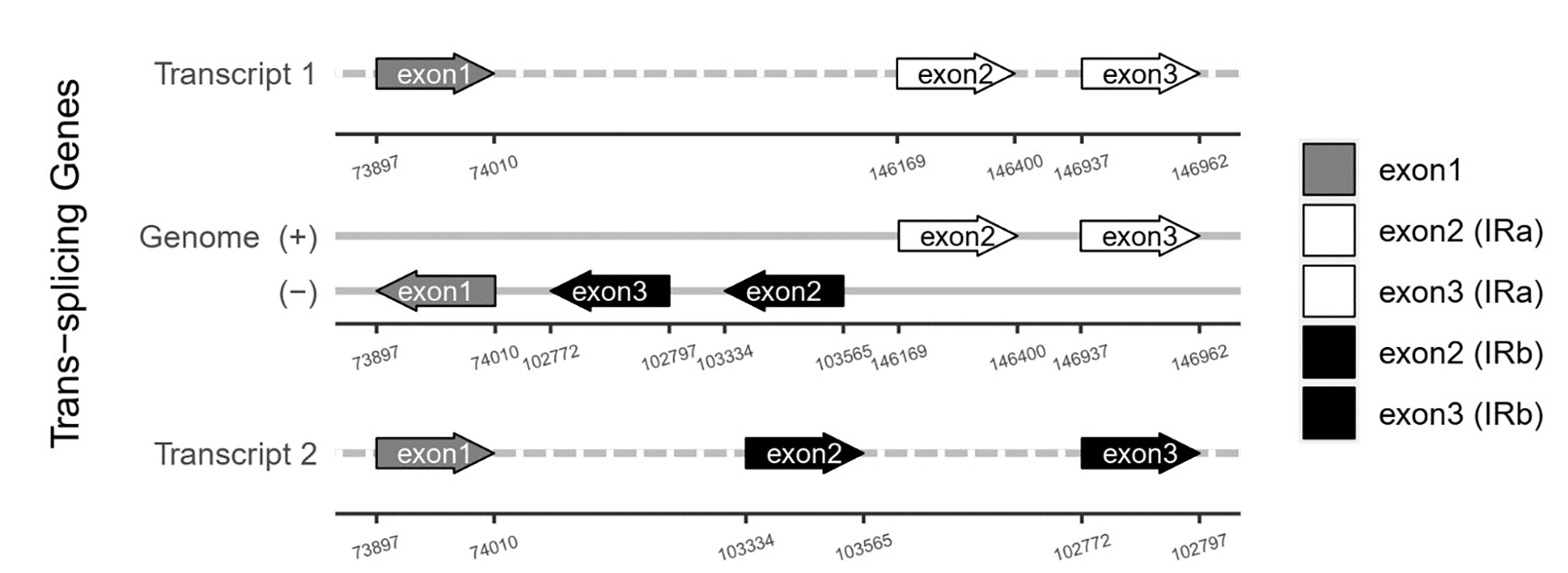

Supplement: Supplemental Material [file TMDN_A_2622795_SM8121.jpg]

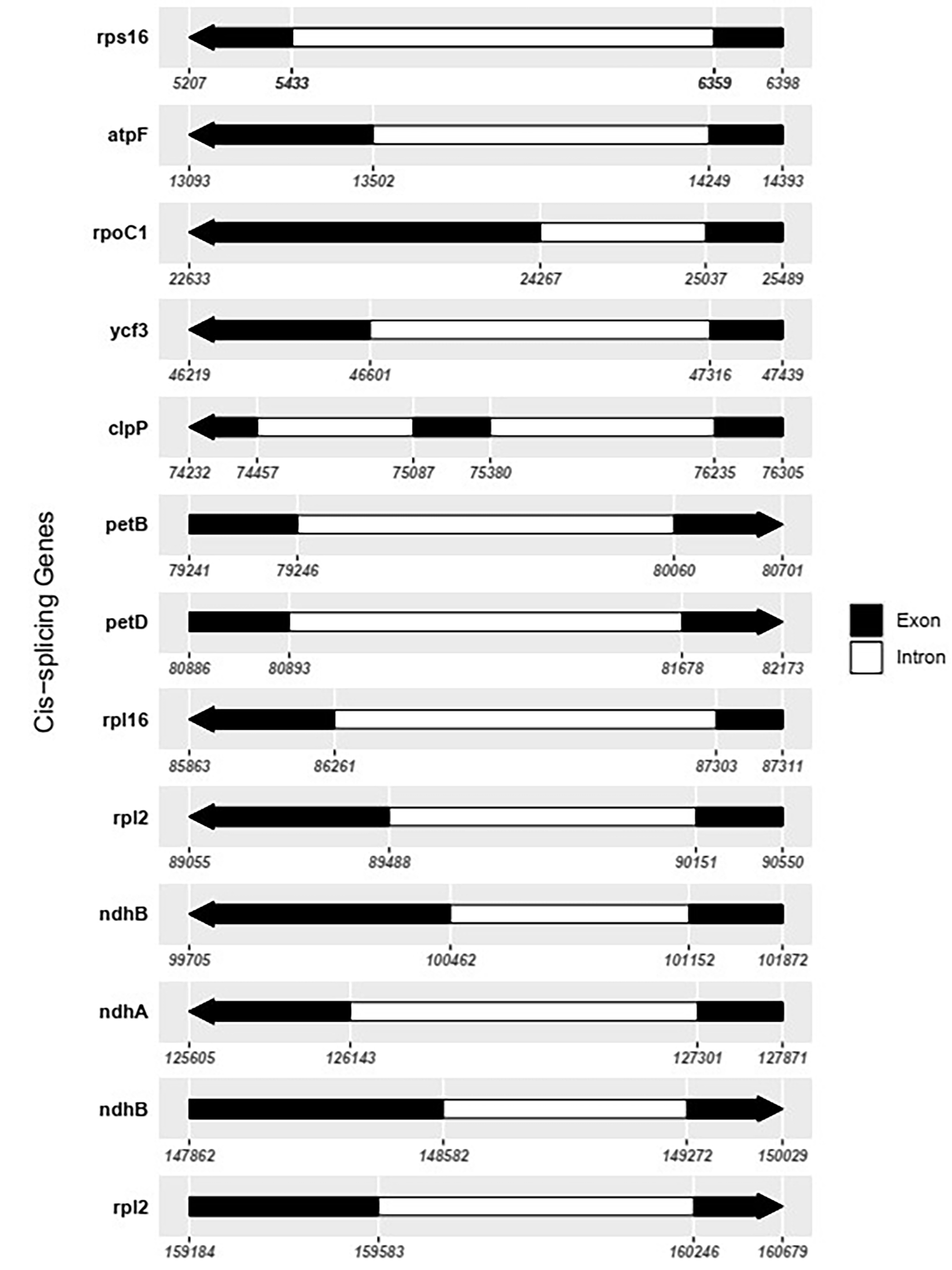

Supplement: Supplemental Material [file TMDN_A_2622795_SM8097.jpg]

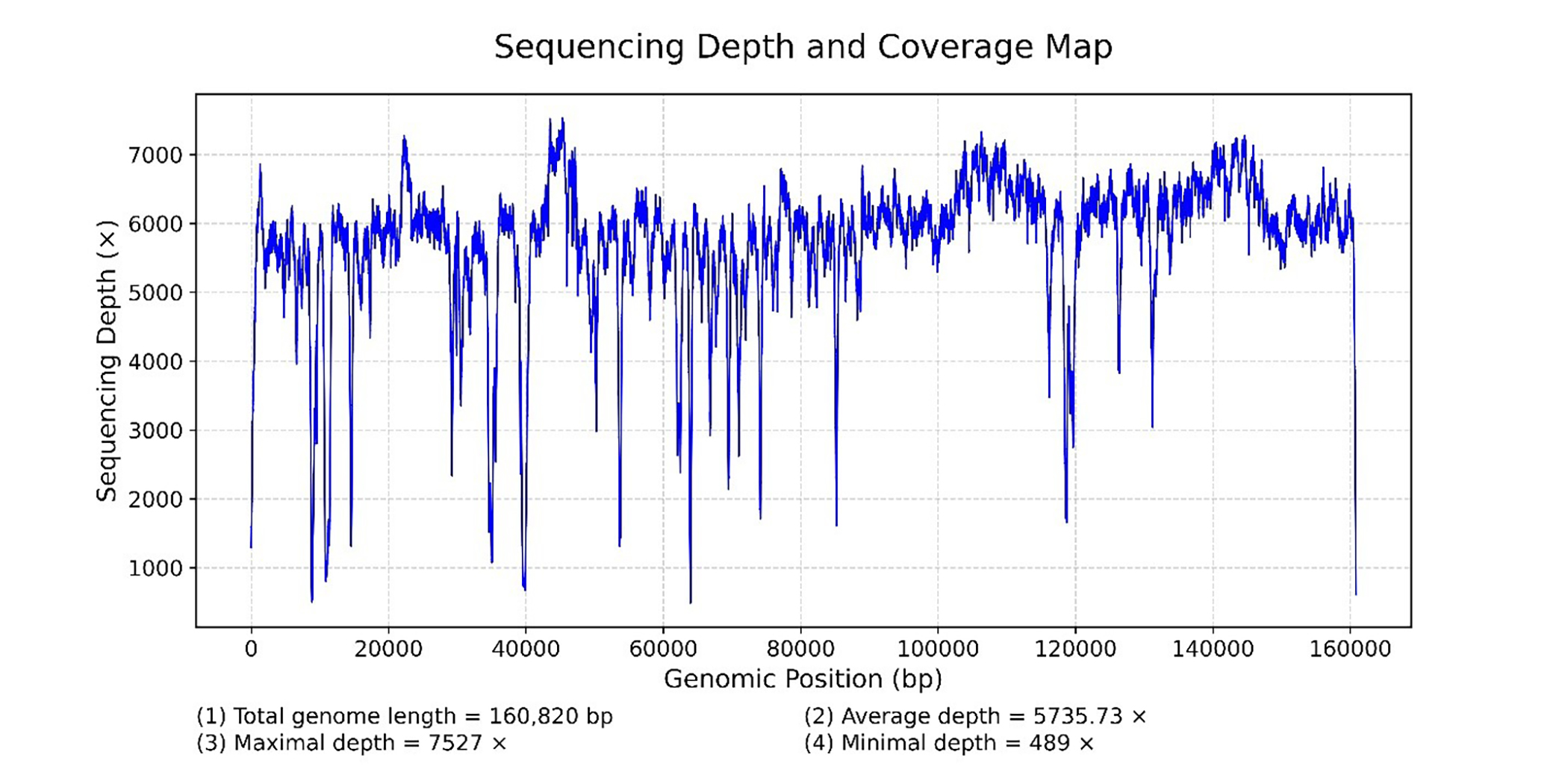

Supplement: Supplemental Material [file TMDN_A_2622795_SM8069.jpg]
